# A Case of a Ruptured Sclerosing Liver Hemangioma

**DOI:** 10.4061/2011/942360

**Published:** 2011-05-31

**Authors:** Haris Papafragkakis, Martin Moehlen, Monica T. Garcia-Buitrago, Beatrice Madrazo, Eddie Island, Paul Martin

**Affiliations:** ^1^Division of Hepatology, University of Miami Miller School of Medicine, 1500 NW 12th Avenue, Suite 1101, Miami, FL 33136, USA; ^2^Department of Pathology, University of Miami Miller School of Medicine, 1611 NW 12th Avenue, Holtz Building, Room 2042, Miami, FL 33136, USA; ^3^Department of Radiology, University of Miami Miller School of Medicine, 1611 NW 12th Avenue, Miami, FL 33136, USA; ^4^Miami Transplant Institute, University of Miami Miller School of Medicine, Highland Professional Building, 1801 NW 9th Avenue, 3rd Floor, Miami, FL 33136, USA

## Abstract

Hemangiomas are the most common benign tumors found in the liver, typically asymptomatic, solitary, and incidentally discovered. Although vascular in nature, they rarely bleed. We report a case of a 52-year-old woman with a previously stable hemangioma who presented to our hospital with signs and symptoms indicative of spontaneous rupture. We review the literature, focusing on diagnosis and management of liver hemangiomas.

## 1. Case

**Figure 1:**
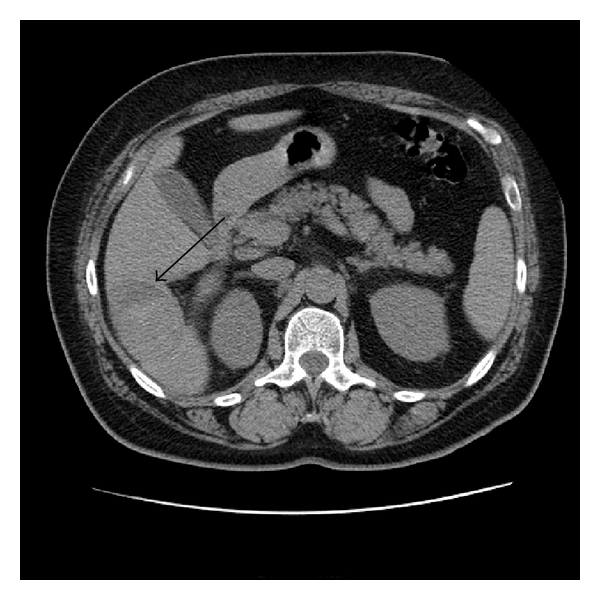
Unenhanced computed tomography of the liver demonstrates a 53.5 mm heterogeneous area in segment six (arrow).

**Figure 2:**
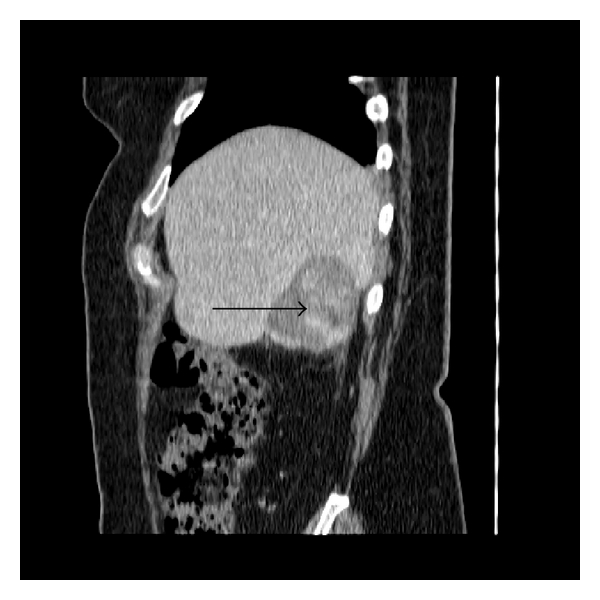
Contrast-enhanced computed tomography of the abdomen in the coronal plane demonstrates intralesional areas of arterial enhancement (arrow). There is a small amount of paracolic gutter fluid.

**Figure 3:**
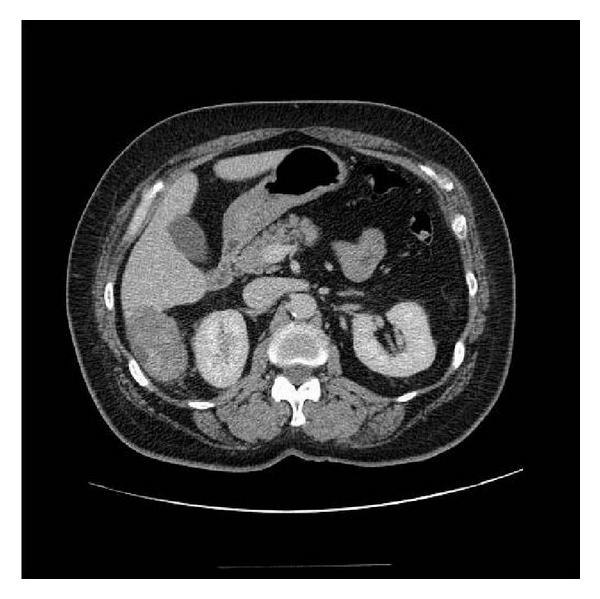
Reimaging of the abdomen with computed tomography three days subsequent to the original study demonstrates further increase in size of the ruptured hemangioma, and it now measures 61.5 mm.

**Figure 4:**
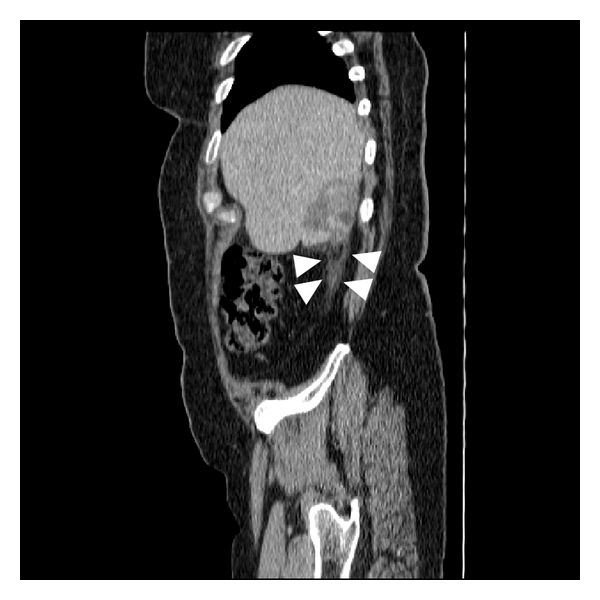
Reimaging of the abdomen with computed tomography three days subsequent to the original study in the coronal plane exhibits an increase in the amount of paracolic gutter fluid (arrowheads), and the craniocaudal dimension of the hemangioma is now 73.8 mm.

**Figure 5:**
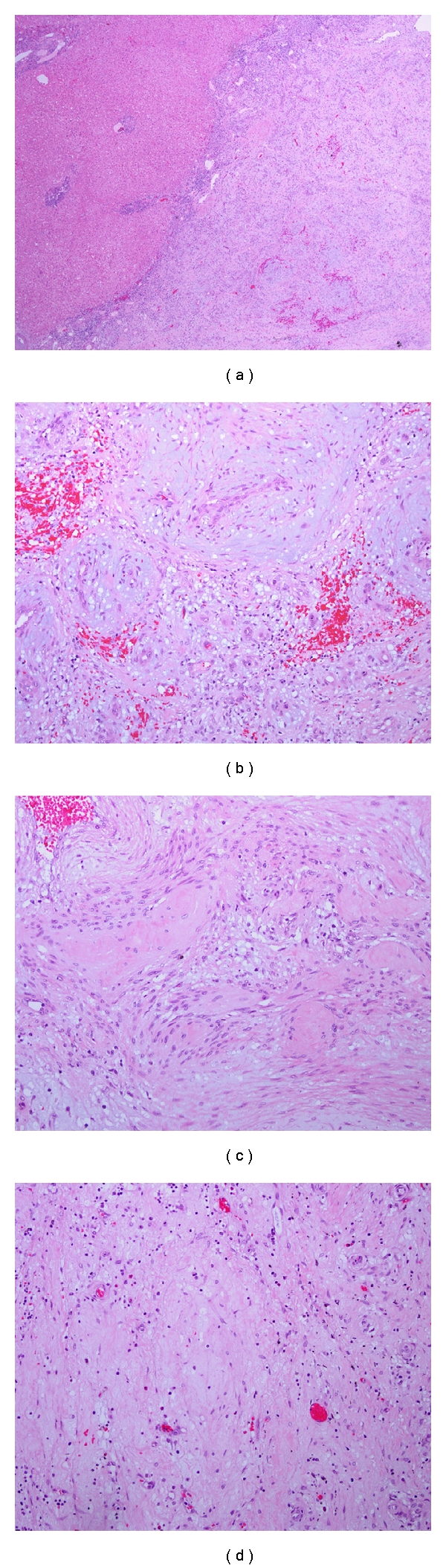
(a) Liver parenchyma with adjacent nonencapsulated mass (H&E, 20x), (b) tumor with a proliferation of thickened blood vessels with myxoid changes (H&E, 400x), (c) the hemangioma shows areas of hyalinized fibrosis (H&E, 400x), and (d) areas of loose connective tissue within the tumor (H&E, 400x).

A 52-year-old Hispanic woman with history of a stable liver mass of undetermined etiology diagnosed over ten years ago presented to our institution with increasing right upper quadrant abdominal pain for one month that had intensified within the last two weeks. Pain intensity was rated 10 out of 10 and increased with deep inspiration. She denied any other associated symptoms, relieving or exacerbating factors. She denied any history of icteric illnesses in the past, any excessive alcohol use, or any parenteral risk factors for viral hepatitis. She had been on an oral contraceptive briefly in her mid-thirties. Her past medical history was negative for parenchymal liver disease but noted for hypertension, type 2 diabetes mellitus, dyslipidemia, obesity, and two previous uncomplicated pregnancies carried to term. Her surgical history was noted for a hysterectomy for dysfunctional uterine bleeding. Her social and family history was unremarkable. On exam, her vital signs were stable and her body mass index was 31. She was anicteric with a regular heart rate. Her lungs were clear, and abdominal exam was noted for right upper quadrant tenderness, nondistended abdomen without a palpable spleen. She had good peripheral pulses and capillary refill, without evidence of edema. Her laboratory investigation revealed an unremarkable complete blood cell count, complete metabolic panel, coagulation studies, and viral hepatitis serologies. A computed tomography of her abdomen revealed a 6.2 × 5.9 × 4.1-centimeter exophytic lesion arising from the posteroinferior aspect of the right liver lobe with a small amount of fluid tracking inferior to the lesion into the right parapelvic gutter suggestive of hemorrhage (Figures 1, 2, 3, and 4). No additional hepatic lesions were identified. There was no radiographic evidence of cirrhosis. She was admitted and underwent laparoscopic resection of the liver lesion. Frank hemoperitoneum was absent at laparotomy. The resected specimen consisted of a 108-gram, 10 × 6 × 3 cm section of liver that contains a 7.5 × 4 × 3.5 cm gray tan, firm, well-circumscribed, nonencapsulated mass with dilated spaces ranging from 0.1 to 0.5 cm. The adjacent liver parenchyma was grossly unremarkable. On histologic examination, the mass showed a proliferation of cavernous vascular spaces with thickened myxoid walls and flattened endothelial lining without cytologic atypia or mitotic activity. The tumor showed extensive areas of dense and loose fibrous tissue made of collagen and elastic fibers, degenerative changes, and hyalinization findings diagnostic of sclerosing (ancient) hemangioma (Figure 5). 

## 2. Discussion

### 2.1. Background

Hemangiomas are the most common benign tumors of the liver, found in one series up to 7% of autopsies [1]. Most hemangiomas are cavernous, in contrast to the peripheral hemangiomas of the capillary type [2, 3]. It is well established that hemangiomas have a female predilection in a ratio of 5 : 1. They are primarily discovered incidentally during abdominal imaging and mainly present in individuals forty to fifty years old [4]. Hemangiomas usually present as solitary lesions, but in approximately 10% of the cases more than one lesion is identified [5, 6]. The pathogenesis of hemangiomas has not been elucidated, but there are two competing theories. The first theory supports the notion that there is overexpression of angiogenic factors such as vascular endothelial growth factor, basic fibroblast growth factor, and metalloproteinases as well as downregulation of some inhibitors of angiogenesis, such as tissue inhibitor of metalloproteinase-I. The second theory is that the presence of liver hemangiomas involves a genetic background of mutations [7–9]. Zhang et al. presumed that metalloproteinases accumulate in the endoplasmic reticulum of the tumor cells, causing self-digestion and vacuole formation [10]. Additionally, Hu et al. showed the cavernous hemangioma cell to downregulate Derlin-1, a protein that when overexpressed induces the dilated endoplasmic reticulum to return to its normal size [11]. Clearly further studies are needed on the pathogenesis of liver hemangiomas and their association with pregnancy, oral contraceptives, and androgen use. 

### 2.2. Diagnosis of Liver Hemangioma, Imaging Modalities, and Role of Biopsy

The imaging modality that is primarily used for the diagnosis of liver hemangiomas is the ultrasound. Characteristically, on ultrasound, the hemangioma is a hyperechoic lesion, predominantly found in the posterior segment of the right liver lobe located inferior to Glisson's capsule, without a peripheral hypoechoic halo and/or hypoechoic center [12, 13]. It is noteworthy that, in patients with fatty liver, hemangiomas can appear hypoechoic and in those with severe fatty infiltration can be hyperattenuated, mimicking hypervascular tumors. Ultrasound with contrast can be used as an alternative to computed tomography (CT) or magnetic resonance imaging (MRI) for undetermined lesions [1]. On triphasic CT, hemangiomas are typically described as having peripheral puddles, filling centripetally, and enhancing in delayed images. MRI is the best, albeit most expensive imaging modality, demonstrating peripheral enhancement, centripetal progression, and hyperintensity on T2 with hypointensity on T1 imaging [14]. Overall, it has been reported that 10% of hemangiomas cannot be reliably diagnosed with imaging methods alone [1, 15]. 

Grossly hemangiomas are described as “spongy” with vascular compartments of various sizes separated by fibrous tissue. Thrombi may be present and are well separated from the normal liver parenchyma despite the absence of a fibrous capsule. They express CD31, CD3 markers, and factor VIII-related antigen among other markers [12]. Hemangioma-like vessels are found extending 1-2 cm around giant (>4 cm in diameter) hemangiomas. Phleboliths and thrombi as well as sclerosis can be found in liver hemangiomas. Sclerosed hemangiomas form, from the center out, have collagenous elastic fibers and are devoid of vascular compartments. The diagnosis of a sclerosed hemangioma is often difficult to make, but is suggested by the presence of small peripheral vessels in the pathology specimen [12]. Given the vascular nature of liver hemangiomas, liver biopsy was in the past considered unsafe and not recommended; however, now it is considered relatively safe to perform, especially when imaging is nondiagnostic [13]. Caution is advised for biopsy of such lesions in question when hepatocellular carcinoma remains in the differential diagnosis and should then only be considered when other methods of diagnosis have been thoroughly exhausted given concerns about seeding the biopsy tract. 

### 2.3. Management of Large and Ruptured Liver Hemangiomas

Liver hemangiomas usually do not cause symptoms unless their diameter exceeds 4 cm. If smaller, they very rarely cause symptoms and almost universally the liver enzymes are within normal limits (unless there is parenchymal liver disease) [12]. The majority of symptomatic patients present with abdominal pain, abdominal fullness, indigestion, and abdominal distention. Anemia, thrombocytopenia, and rare syndromes such as the Kasabach-Merritt syndrome (a consumptive coagulopathy) or Bornman-Terblanche-Blumgart syndrome (fever and abdominal pain) have been reported [3, 14, 16]. Rupture of liver hemangiomas is very rare [17, 18]. The first case was reported by Sewell [3] and Haefen [19]. There are few cases of hemangioma rupture reported in the literature; the vast majority of giant hemangiomas are larger than 4 cm in diameter, some of which presented with rupture during pregnancy, when hemangiomas characteristically increase in size. In our patient, the hemangioma apparently ruptured but without causing hemodynamic instability. Perhaps, as this hemangioma was sclerosed on histological examination, it may have contributed to the subacute presentation of our patient. 

There are a number of interventions used to treat symptomatic hemangiomas. The first report of surgery for a hemangioma was by Karp in 1931. In a study from Mayo clinic including 49 cases of hemangiomas exceeding 4 cm in diameter, 13 patients underwent surgery, from simple excision to hepatic lobectomy and 36 other patients were observed for 15 years [20]. Rupture was not observed in the latter group. Despite this, Corigliano et al. suggested that hemangiomas with a diameter greater than 10 cm have a higher risk for internal bleeding, growth, and rupture, and therefore preventive excision is recommended even if asymptomatic [18]. The Pringle maneuver, enucleation with temporarily inflow occlusion, is considered to be the treatment of choice for large hemangiomas due to less blood loss and postoperative complications [21]. Complications may include infection, sepsis, fluid collection around the liver, and paralytic ileus secondary to abdominal fluid collection [22]. Embolization is another means of treating a symptomatic or very large lesion, and it should be targeted at the hepatic artery branches that feed the lesion [23–25]. The most common complications of embolization are abdominal pain, fever, and nausea, with very rare complications including sepsis and migration of the thrombotic material. It has been recommended that embolization can be performed prior to resection of giant hemangiomas in order to diminish future complications and reduce intraoperative blood loss. Other less common treatment modalities include radiation therapy and liver transplantation [22, 26, 27]. 

We reported a case of a ruptured giant hepatic hemangioma in a nonpregnant woman that was managed with hepatic resection. In the literature, there are few reported cases of spontaneously ruptured liver hemangiomas, mostly cavernous type, most of which were treated urgently with resection or transarterial embolization due to extensive hemoperitoneum and hemodynamic instability. In our case, the patient presented and remained in a stable condition throughout the hospitalization until her surgery. Despite having imaging findings of giant hemangioma rupture, she never became hemodynamically unstable. We could not identify cases related to spontaneous rupture of a sclerosing, (ancient) liver hemangioma. A PubMed search of the English literature with the keywords “spontaneous rupture, hepatic-liver and sclerosed or sclerosing hemangioma,” yielded no results. Of course, the lack of references on spontaneous sclerosing liver hemangioma rupture may be partially due to insufficient or no reporting of pathology findings in some of the published cases. Our case suggests that spontaneous rupture may occur in a cavernous liver hemangioma with abundant sclerosing features; however, this may be responsible for a subacute presentation, not significant hemoperitoneum and no hemodynamic instability, as happened with our patient. In conclusion, a hemangioma is the most common benign lesion found in the liver. Although typically an incidental finding, discussed potentially severe complications can occur requiring prompt intervention.
